# Glutamine is a substrate for glycosylation and CA19-9 biosynthesis through hexosamine biosynthetic pathway in pancreatic cancer

**DOI:** 10.1007/s12672-023-00628-z

**Published:** 2023-02-17

**Authors:** Chen Liu, Shengming Deng, Zhiwen Xiao, Renquan Lu, He Cheng, Jingjing Feng, Xuxia Shen, Quanxing Ni, Weiding Wu, Xianjun Yu, Guopei Luo

**Affiliations:** 1 Department of Pancreatic Surgery, Fudan University Shanghai Cancer Center, No. 270,Dong’An Rd, Xuhui District, Shanghai, 200032 China; 2 Department of Oncology, Shanghai Medical College, Fudan University, Shanghai, 200032 China; 3 Pancreatic Cancer Institute, Fudan University, Shanghai, 200032 China; 4 Shanghai Pancreatic Cancer Institute, Shanghai, 200032 China; 5 Department of Clinical Laboratory, Fudan University Shanghai Cancer Center, Shanghai, 200032 China; 6 Department of Pathology, Fudan University Shanghai Cancer Center, Shanghai, 200032 China

**Keywords:** Pancreatic adenocarcinoma, Glutamine, CA19-9, GlcNAc, Glucose

## Abstract

**Background:**

Carbohydrate antigen 19–9 (CA19-9) is the most widely used biomarker for pancreatic cancer. Since CA19-9 closely correlates with patient outcome and tumor stage in pancreatic cancer, the deciphering of CA19-9 biosynthesis provides a potential clue for treatment.

**Methods:**

Concentration of amino acids was detected by ultrahigh-performance liquid chromatography tandem mass spectrometry. Metabolic flux of glutamine was examined by isotope tracing untargeted metabolomics. Label-free quantitative n-glycosylation proteomics was used to examine n-glycosylation alterations.

**Results:**

Among all amino acids, glutamine was higher in CA19-9-high pancreatic cancers (> 37 U/mL, 66 cases) than in CA19-9-normal clinical specimens (≤ 37 U/mL, 37 cases). The glutamine concentration in clinical specimens was positively correlated with liver metastasis or lymphovascular invasion. Glutamine blockade using diazooxonorleucine suppressed pancreatic cancer growth and intraperitoneal and lymphatic metastasis. Glutamine promotes O-GlcNAcylation, protein glycosylation, and CA19-9 biosynthesis through the hexosamine biosynthetic pathway. UDP-n-acetylglucosamine (UDP-GlcNAc) levels correlated with the glutamine influx through hexosamine biosynthetic pathway and supported CA19-9 biosynthesis.

**Conclusions:**

Glutamine is a substrate for CA19-9 biosynthesis in pancreatic cancer. Glutamine blockade may be a potential therapeutic strategy for pancreatic cancer.

## Introduction

Pancreatic ductal adenocarcinoma (PDAC), a major form of pancreatic cancer, is a highly lethal carcinoma with a mortality almost equal to its incidence [1]. There are no methods for early detection; as a result, only approximately 20% of patients with pancreatic cancer have the opportunity to receive surgical resection, which provides the only chance of cure. Although major advances in cancer treatment have been made in the recent past, the outcome of pancreatic cancer has only modestly improved [2].

A distinct pathologic trait for pancreatic cancer is extensive desmoplasia, which accounts for approximately 80% of the tumor mass [3]. There is still an intense dispute concerning whether stroma promotes or prevents the development of pancreatic cancer [3]. An interconversion between collagen and glutamine through proline is well recognized, and the direction mainly depends on the tumor microenvironment and genetic alterations such as *MYC* and *KRAS* [4].

Sialyl Lewis a (CA19-9), also called carbohydrate antigen 19–9 is the most widely used and best validated biomarker for the management of pancreatic cancer [5]. Approximately 80% of pancreatic cancer patients have elevated levels of circulating CA19-9, and the level of CA19-9 is positively correlated with tumor burden and stage. As a biomarker, CA19-9 could be employed to evaluate tumor stage, assess prognosis and resectability, and monitor therapeutic response [5]. Recent studies have shown that CA19-9 is not merely a “bystander” but may actively participate in the progression of pancreatic cancer [6, 7].

Metabolic reprogramming has long been recognized as an emerging hallmark of cancer [8]. Glutamine, a nonessential amino acid, is the most abundant amino acid in the circulation of the human body [9]. Most types of cancer have a strong metabolic dependency to glutamine for development [10]. Glutamine is a super nutrient that supplies fuel for energy production and biosynthesis of lipids, glucose, and nucleotides mainly through the tricarboxylic acid (TCA) cycle [10, 11]. Pancreatic cancer cells, which reside in a harsh and nutrient-poor microenvironment, have a particular requirement for glutamine for progression [8, 12]. However, the connections between glutamine and clinical relevance or CA19-9 production have not been explored.

## Results

### Glutamine levels are positively correlated with aggressive clinical characteristics and CA19-9

**Fig. 1:**
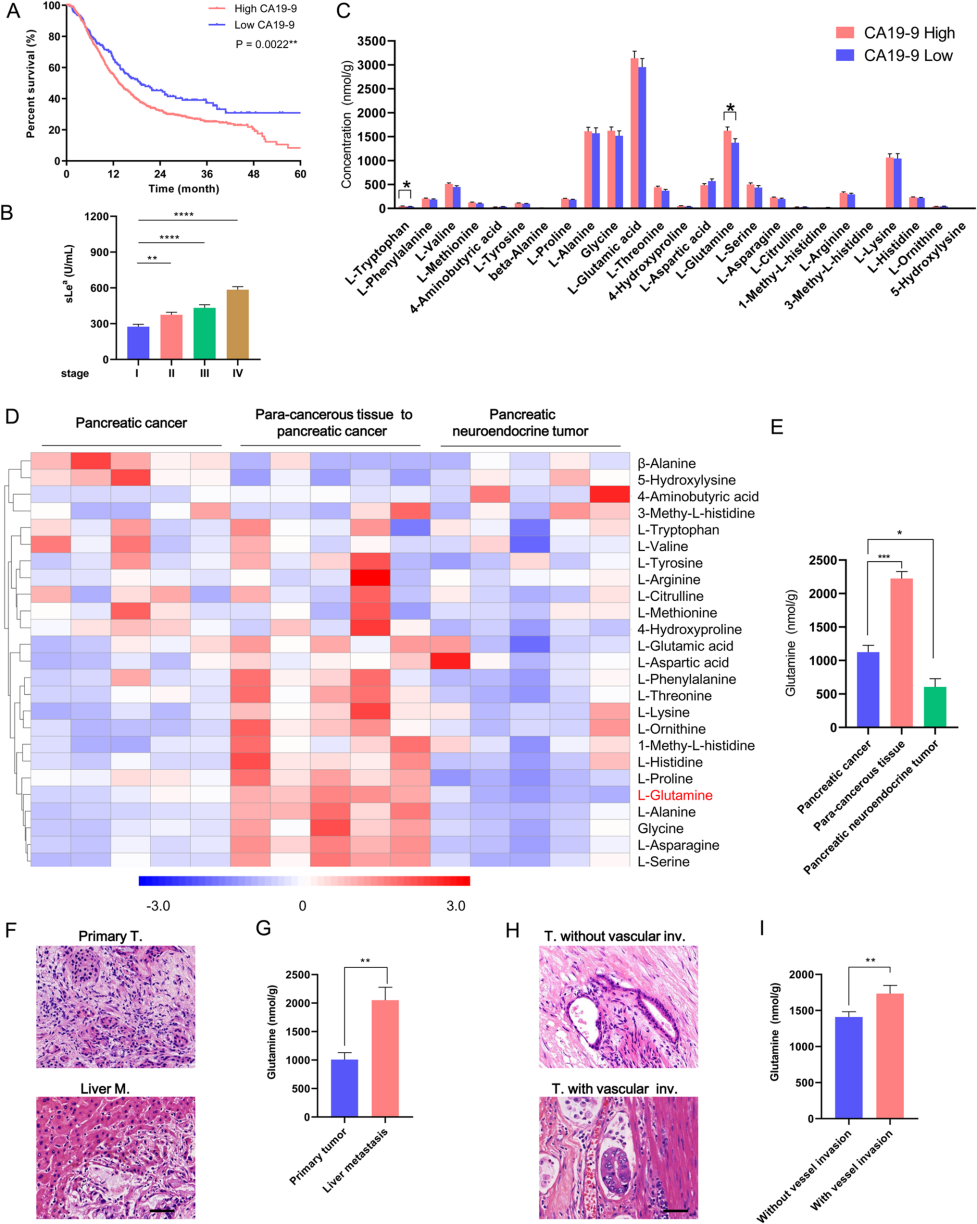
Glutamine levels are positively correlated with aggressive clinical characteristics in pancreatic cancer. **A** Kaplan‒Meier overall survival curve for patients with pancreatic cancer divided by baseline serum CA19-9 levels. CA19-9-low, ≤ 37 U/mL, 224 cases; CA19-9–9-high, > 37 U/mL, 844 cases. **B** The serum concentration of CA19-9 for patients with pancreatic cancer categorized using the American Joint Committee on Cancer 8^th^ staging system. **C** Quantification of amino acid concentration by UHPLC‒MS/MS in resected pancreatic cancers stratified by serum CA19-9 level (low, ≤ 37 U/mL, 37 cases; high, > 37 U/mL, 66 cases). UHPLC–MS/MS, ultrahigh-performance liquid chromatography tandem mass spectrometry. **D**, **E** Heatmap of amino acids in pancreatic cancers, tissue adjacent to pancreatic cancer, and pancreatic neuroendocrine tumors detected by UHPLC‒MS/MS (**D**). Values are the log 10 transformed of the concentration of amino acids. Quantification of glutamine concentration in pancreatic cancer, tissue adjacent to pancreatic cancer, and pancreatic neuroendocrine tumors (5 cases for each group, E) are shown. **F**, **G** Representative hematoxylin and eosin (H&E) staining images of primary pancreatic cancers and their matched liver metastases (6 cases, F). Quantification of glutamine concentration in primary pancreatic cancers and their matched liver metastases (**G**). **H**, **I** Representative H&E staining images of primary pancreatic cancers with (36 cases) or without (57 cases, H) lymphovascular invasion. Quantification of glutamine concentration in resected pancreatic cancer stratified by vascular invasion status (**I**). Scale bars, 50 μm. Data represent the mean ± s.e.m., *p < 0.05; **p < 0.01; ***p < 0.001; ****p < 0.0001

CA19-9 has been reported to be the best-validated biomarker in pancreatic cancer, which is closely correlated with patient outcome and tumor stage [5, 13]. By analyzing 1028 patients with pancreatic cancer, we confirmed that CA19-9 elevation was an adverse prognostic predictor (Fig. 1A) and that the level of CA19-9 was positively correlated with tumor stage (Fig. 1B). To screen amino acids that may play a key role in tumor progression and CA19-9 biosynthesis, the concentration of amino acids in clinical specimens was analyzed by ultrahigh-performance liquid chromatography tandem mass spectrometry (UHPLC–MS/MS). Glutamine was higher in CA19-9-high pancreatic cancers (> 37 U/mL, 66 cases) than in CA19-9-low specimens (≤ 37 U/mL, 37 cases; Fig. 1C). The level of glutamine in pancreatic cancers was lower than that in paracancer tissues and higher than that in pancreatic neuroendocrine neoplasms (Fig. 1D, E). This observation indicates that paracancer tissues may provide glutamine for pancreatic cancer cells progression. Interestingly, primary pancreatic cancers had a lower concentration of glutamine than their matched liver metastases (Fig. 1F, G). Moreover, glutamine was more abundant in pancreatic cancers with lymphovascular invasion than in those without (Fig. 1H, I). These findings confirm that pancreatic cancer cells have strong dependence on glutamine for growth/invasion/metastasis and are consistent with the observation that the concentration of glutamine in pancreatic cancer was correlated positively with CA19-9. Taken together, these results indicate that glutamine is positively correlated with aggressive clinical characteristics in pancreatic cancer.

### Glutamine promotes pancreatic cancer growth and metastasis

**Fig. 2:**
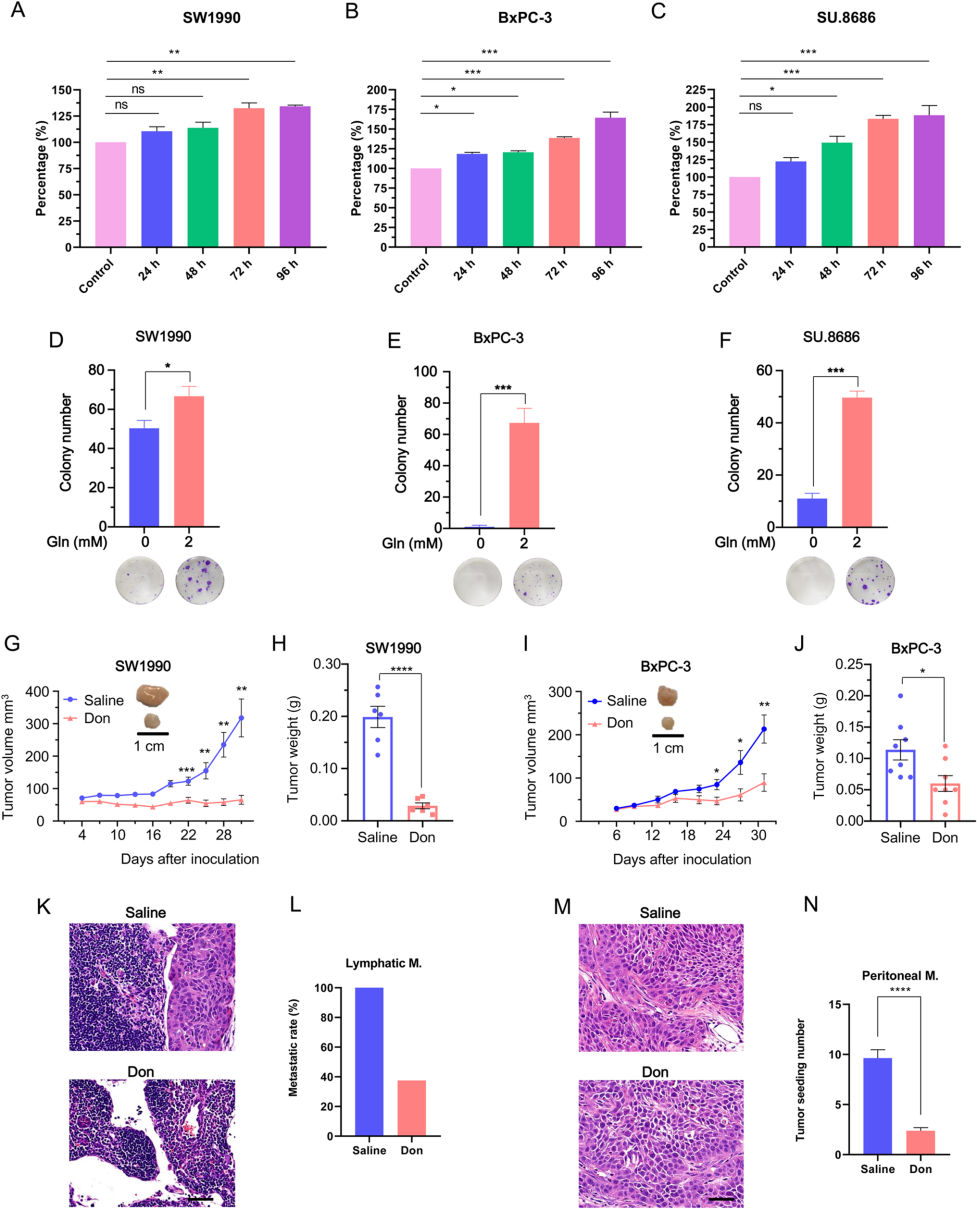
Glutamine promotes pancreatic cancer progression. **A–C** The proliferation of SW1990 (**A**), BxPC-3 (**B**), and SU.8686 (**C**) cells cultured in 2 mM glutamine containing media were compared with cells cultured in glutamine-free media using Cell Counting Kit-8. **D–F** Colony formation ability of SW1990 (**D**), BxPC-3 (**E**), and SU.8686 (**F**) cells cultured in 2 mM glutamine containing media were compared with cells cultured in glutamine-free media. Quantification (above) and representative images (below) are shown. **G**, **H** Tumor growth (**G**) and weight (**H**) of SW1990 cells treated with or without Don in BALB/c nude mice. Tumor growth was measured at the indicated time points and dissected at the endpoint. n = 6 mice per group. **I**, **J** Tumor growth (**I**) and weight (**J**) of BxPC-3 cells treated with or without Don in BALB/c nude mice. n = 8 mice per group. **K**, **L** Lymphatic metastatic rate of BxPC-3 footpad mouse models treated with (37.5%) or without Don (100.0%) in BALB/c nude mice. n = 9 mice per group. **M**, **N** Peritoneal seeding of BxPC-3 intraperitoneal mouse models treated with or without Don in BALB/c nude mice. n = 9 mice per group. Don, diazooxonorleucine. Scale bars, 50 μm. Data represent the mean ± s.e.m., *p < 0.05; **p < 0.01; ***p < 0.001; ****p < 0.0001

Pancreatic cancer cells have a strong need for glutamine, which is a substrate to support anabolic processes that fuel proliferation [14]. Moreover, glutamine metabolism also plays an active role in the invasive potential of pancreatic cancer [15]. In this study, the proliferation of pancreatic cancer cells (SW1990, BxPC-3, and SU.8686) at different time points with and without 2 mM glutamine was determined by CCK-8 and colony formation assays. Glutamine promoted the proliferation of all pancreatic cancer cells (Fig. 2A, C). Moreover, glutamine enhanced colony formation in different pancreatic cancer cells (Fig. 2D–F). Colonies were less formed in pancreatic cancer cells cultured for two weeks in the absence of glutamine. Therefore, glutamine is an essential nutrient for the progression of pancreatic cancer. Diazooxonorleucine (Don), an inhibitor of glutamine, inhibited tumor growth (Fig. 2G–J) in subcutaneous mouse models of pancreatic cancer. Don also suppressed lymphatic metastasis (Fig. 2K, L) in footpad mouse models and peritoneal seeding (Fig. 2M, N) in peritoneal mouse models. The decrease in metastasis may be a result of the inhibition of the growth and viability of pancreatic cancer cells using Don. Thus, glutamine not only provides substrates for biosynthesis but also contributes to the metastasis of pancreatic cancer cells.

### Metabolic flux of glutamine in pancreatic cancer cells

**Fig. 3:**
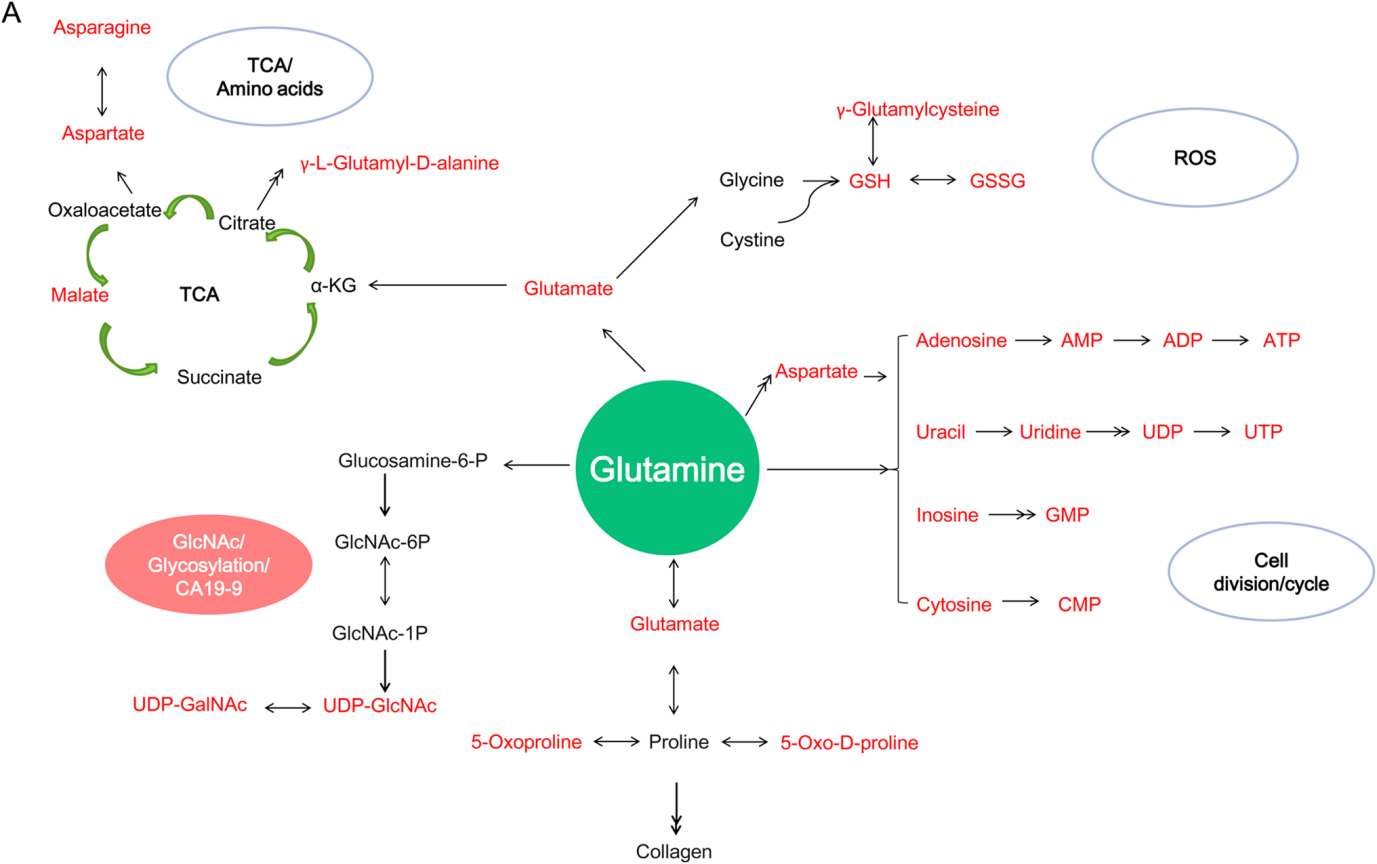
Metabolic flux of glutamine in pancreatic cancer cells. **A** Main flow includes CA19-9 biosynthesis/glycosylation, collagen/stroma, TCA cycle, cell division, and ROS reaction detected by isotope tracing untargeted metabolomics using UHPLC-MS. Three replicates per condition. The double arrow represents multiple reactions using [U-13^C^]-L-glutamine or [U-15^N^]-L-glutamine as tracers. Blue circle, [U-13^C^]-L-glutamine; red circle, [U-15^N^]-L-glutamine. Red indicates metabolites have been traced by isotope. UHPLC-MS, ultrahigh performance liquid chromatography coupled with tandem Q-Exactive Orbitrap mass spectrometer. α-KG, α-ketoglutarate; TCA cycle, tricarboxylic acid cycle; ROS, reactive oxygen species; CA19-9, sialyl Lewis a; UMP, uridine monophosphate; UDP, uridine 5’-diphosphate; UTP, uridine 5’-triphosphate; ADP, adenosine 5’-diphosphate; ATP, adenosine 5’-triphosphate; AMP, adenosine 5’-monophosphate; CMP, cytidine-5’-monophosphate; GMP, guanosine 5’-phosphate; GlcNAc-6P, N-acetylglucosamine-6-phosphate; GlcNAc-1P, N-acetylglucosamine-1-phosphate; UDP-GlcNAc, UDP-N-acetylglucosamine; *UDP-GalNAc* UDP-N-acetyl-D-galactosamine, *fructose-6P*, fructose 6-phosphate, *GSH* reduced glutathione, GSSG glutathione disulfide

To examine the metabolic flux of glutamine in pancreatic cancer cells, C or N isotope labelled glutamine was traced in SU.8686 cells cultured for 12 h and 24 h by isotope tracing untargeted metabolomics using ultrahigh-performance liquid chromatography coupled with tandem Q-Exactive Orbitrap mass spectrometry (UHPLC-MS; Fig. 3A). In addition to its well-recognized role in the TCA cycle, cell division, and reactive oxygen species (ROS) reactions, glutamine also contributes to the biosynthesis of UDP-N-acetylglucosamine (UDP-GlcNAc) and UDP-N-acetyl-D-galactosamine (UDP-GalNAc), critical substrates for glycosylation. Moreover, isotopes were detected in proline, 5-oxoproline, and 5-oxo-D-proline, which have a close relationship with collagen and stroma formation. Notably, the metabolic flow of glutamine in cancer cells is influenced by the tumor microenvironment. In a *MYC*-inducible human Burkitt lymphoma model P493 cell line, glutamine entered the TCA cycle and contributed to citrate carbons under hypoxia and to fumarate, malate, and citrate under glucose deprivation [16].

### Glutamine promotes O-GlcNAcylation

**Fig. 4:**
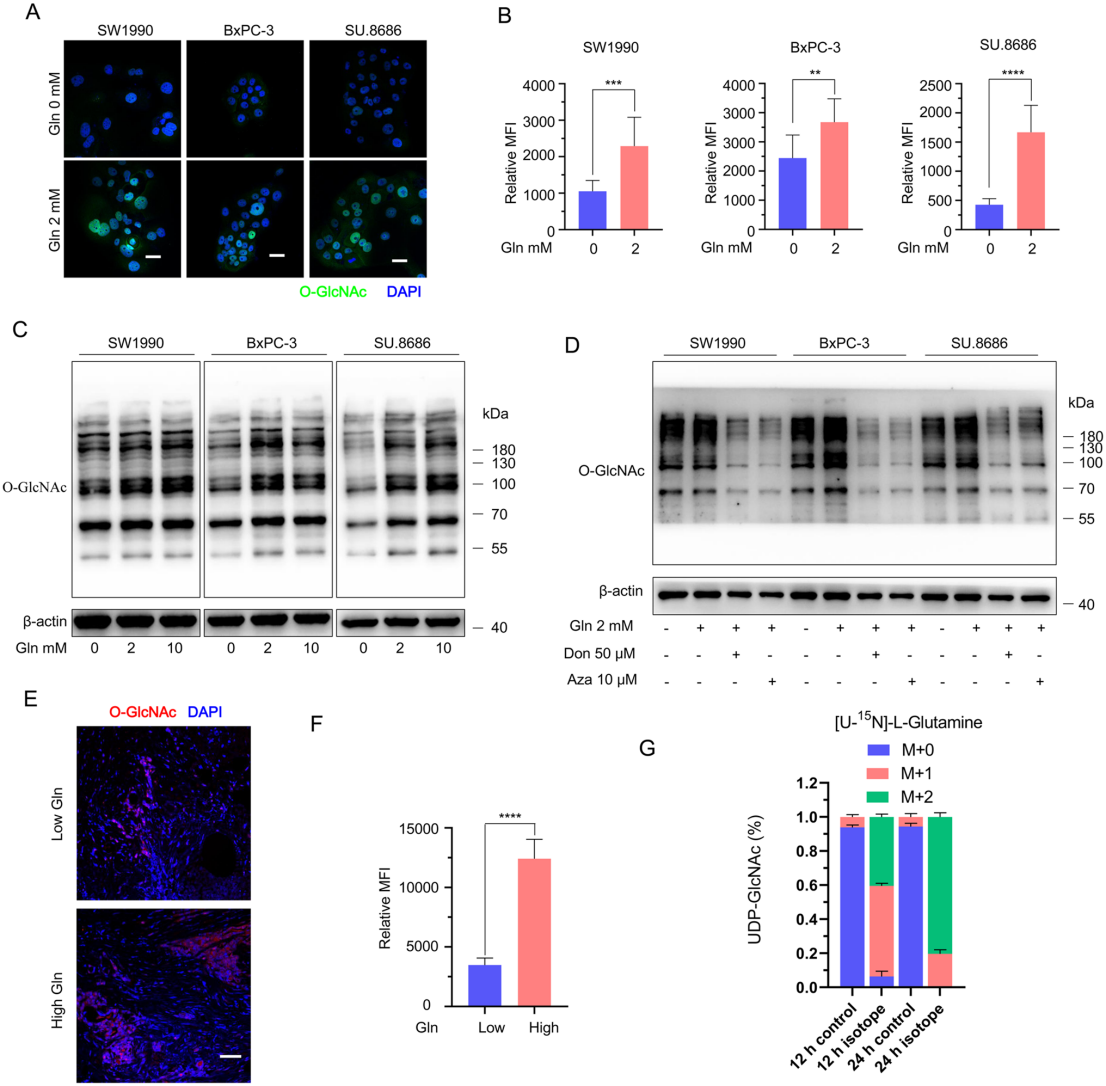
Glutamine promotes O-GlcNAc biosynthesis. **A, B** The addition of 2 mM glutamine to glutamine-free medium increased the expression of O-GlcNAc in SW1990, BxPC-3, and SU.8686 cells, as shown by immunofluorescence confocal microscopy. Cells were cultured in different media for 72 h. Quantification is shown to the right. Data were randomly chosen in seven different fields. Scale bars, 25 μm. **C** The expression of O-GlcNAc in cells cultured in medium containing various concentrations (0, 2, 10 mM) of glutamine for 72 h was examined by Western blot. **D** The expression of O-GlcNAc in cells cultured for 72 h in glutamine-free medium, 2 mM glutamine medium, and 2 mM glutamine medium treated with 50 µM Don or 10 µM Aza was examined by Western blot. **E**, **F** Representative image (**E**) and quantification (**F**) of the expression of O-GlcNAc in high-glutamine pancreatic cancers versus low-glutamine pancreatic cancers as observed by immunofluorescence in clinical samples. n = 6 cases in each group. Scale bars, 50 μm. **G** Fractional isotopomer labelling of UDP-GlcNAc from [U-^15^N]-L-glutamine after 12 h and 24 h using UHPLC-MS. All data are presented as the mean ± s.e.m. M + (M0, M1 to Mn) represents the number of C or N in metabolites labeled by isotopes. The fractional abundance of mass isotopomers defined by M0, M1, and Mn was calculated. Three replicates per condition*. UHPLC-MS* ultrahigh performance liquid chromatography coupled with tandem Q-Exactive Orbitrap mass spectrometer

Glutamine acts as a major substrate for the hexosamine biosynthetic pathway (HBP), an important branch of glycolysis [10]. UDP-GlcNAc, the end-product of HBP, is a critical metabolite used for O-GlcNAcylation as well as for O- and N-glycosylation [17]. O-GlcNAcylation is the covalent attachment of GlcNAc to serine or threonine residues of nuclear and cytoplasmic proteins and is an important posttranslational modification [18]. It plays a key role in cancer-related activities, such as tumor proliferation, cell signaling, metabolism, invasion and metastasis [18]. In this study, we demonstrated that glutamine enhanced O-GlcNAcylation in pancreatic cancer cells (SW1990, BxPC-3, SU.8686) using immunofluorescence confocal microscopy (Fig. 4A, B). The effect of glutamine promoting O-GlcNAc biosynthesis was confirmed using western blotting after cells were cultured in different concentrations of glutamine (Fig. 4C). Glutamine inhibitors (Don and azaserine) decreased the biosynthesis of O-GlcNAc in pancreatic cancer cells (Fig. 4D). The correlation between glutamine and O-GlcNAc expression was confirmed in clinical pancreatic cancer samples (Fig. 4E, F). Isotope labeled glutamine could be traced in UDP-GlcNAc using UHPLC-MS (Fig. 4G). Thus, glutamine promotes O-GlcNAc biosynthesis in pancreatic cancer.

### Glutamine enhances glycosylation

**Fig. 5:**
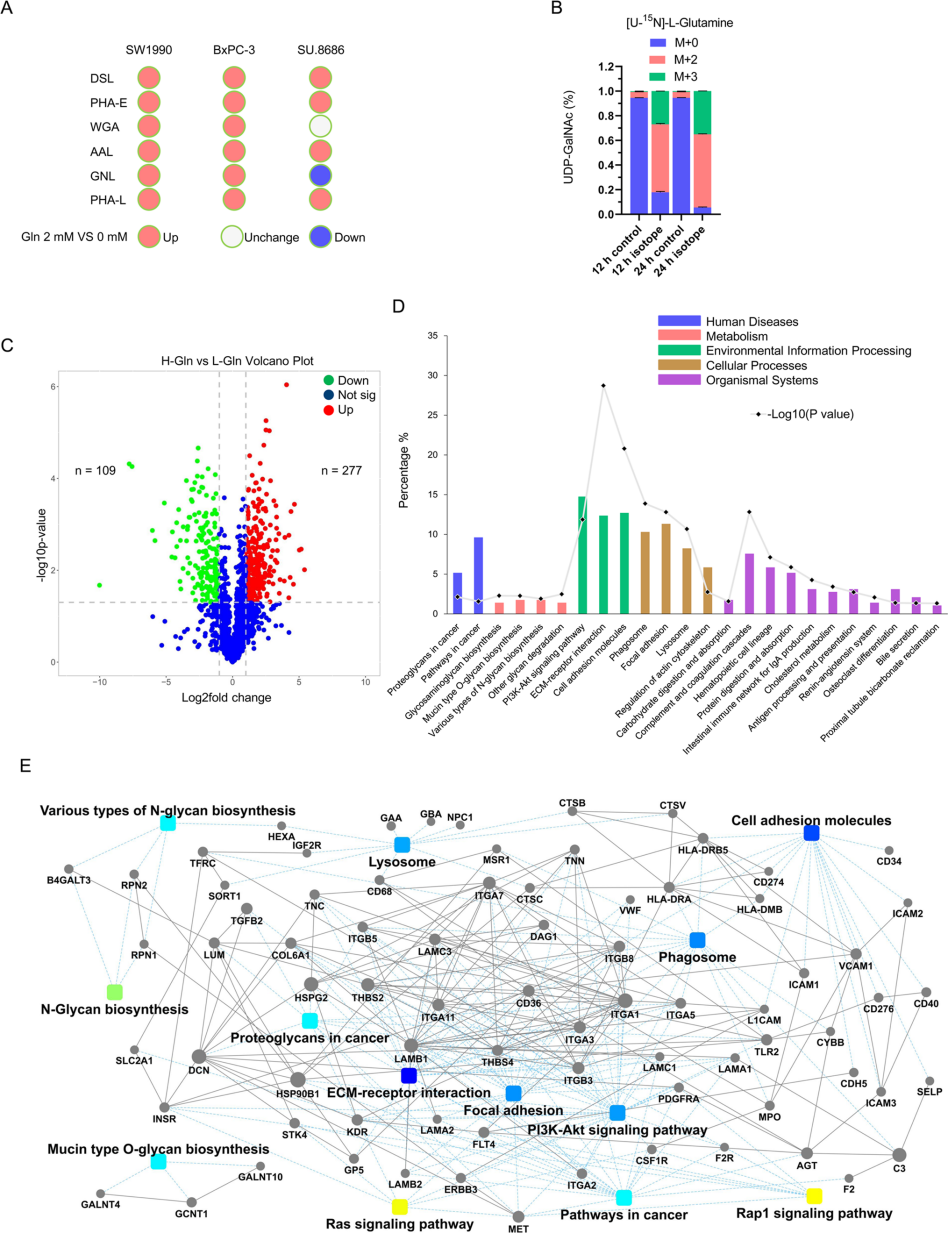
Glutamine enhances N glycosylation. **A** Lectin blot analysis showed the change in lectins in pancreatic cancer cells with the addition of 2 mM glutamine to the culture medium. Lectins, including DSL, PHA-E, AAL, and PHA-L, were upregulated in cells cultured in 2 mM glutamine-containing media compared with cells cultured in glutamine-free media. DSL, GlcNAc/LacNAc; PHA-E, Galβ4GlcNAcβ2; WGA, Neu5Ac, GlcNAc; AAL, fucose; GNL, mannose; PHA-L, Galβ4GlcNAcβ6. **B** Fractional isotopomer labelling of UDP-GalNAc from [U-^15^N]-L-glutamine (**B**) after 12 h and 24 h using UHPLC-MS. M + (M0, M1 to Mn) represents the number of C or N in metabolites labeled by isotopes. The fractional abundance of mass isotopomers defined by M0, M1, and Mn was calculated. Three replicates per condition. UHPLC-MS, ultrahigh performance liquid chromatography coupled with tandem Q-Exactive Orbitrap mass spectrometer. **C–E**, Volcano plot (**C**), Kyoto Encyclopedia of Genes and Genomes (KEGG) pathway (**D**), and protein‒protein interaction (PPI) (**E**) analyses showed N-glycosylation alterations by comparing high glutamine pancreatic cancers (3 samples) with low glutamine pancreatic cancers (3 samples) examined by label-free quantitative glycosylation proteomics using UHPLC‒MS/MS. *UHPLC‒MS/MS* ultrahigh-performance liquid chromatography tandem mass spectrometry

Glycosylation, a process related to the production of glycosidic linkages of saccharides to proteins, lipids, or other saccharides, is widely present in normal cells and regulates fundamental physiological processes [19]. Compared with their non-transformed counterparts, cancer cells generally augment the degree of glycosylation. The function of glycosylation in cancers includes tumor proliferation, invasion and metastasis, cell‒cell communication, signal transduction, and extracellular matrix formation [20]. Aberrant N-linked glycosylation is quite apparent in pancreatic cancer and can be observed by the most widely used serological marker of CA19-9, which contains a glycan known as sialyl Lewis a. In this study, lectin blotting showed that DSL, PHA-E, AAL, and PHA-L were upregulated in cells cultured with glutamine-containing media (Fig. 5A). In addition to UDP-GlcNAc, UDP-GalNAc, an interconverted product of UDP-GlcNAc, could be traced by UHPLC-MS when cells were cultured in isotope-labeled glutamine (Fig. 5B), suggesting that glutamine is a substrate for glycosylation. By comparing N-glycosylation alterations between high- and low-glutamine pancreatic cancers in clinical specimens using label-free quantitative proteomics, cancer-related pathways, including PI3K-Akt signaling, ECM-receptor interaction, and cell adhesion, were identified (Fig. 5C, D). Protein‒protein interactions, including TGFB2, SLC2A1, INSR, LAMB1, ITGA2, ITGA3, ICAM1, CD274, THBS4, and CD36, were observed (Fig. 5E). Taken together, glutamine exacerbated glycosylation modification in pancreatic cancer cells.

### Glutamine is a substrate for CA19-9 biosynthesis

**Fig. 6:**
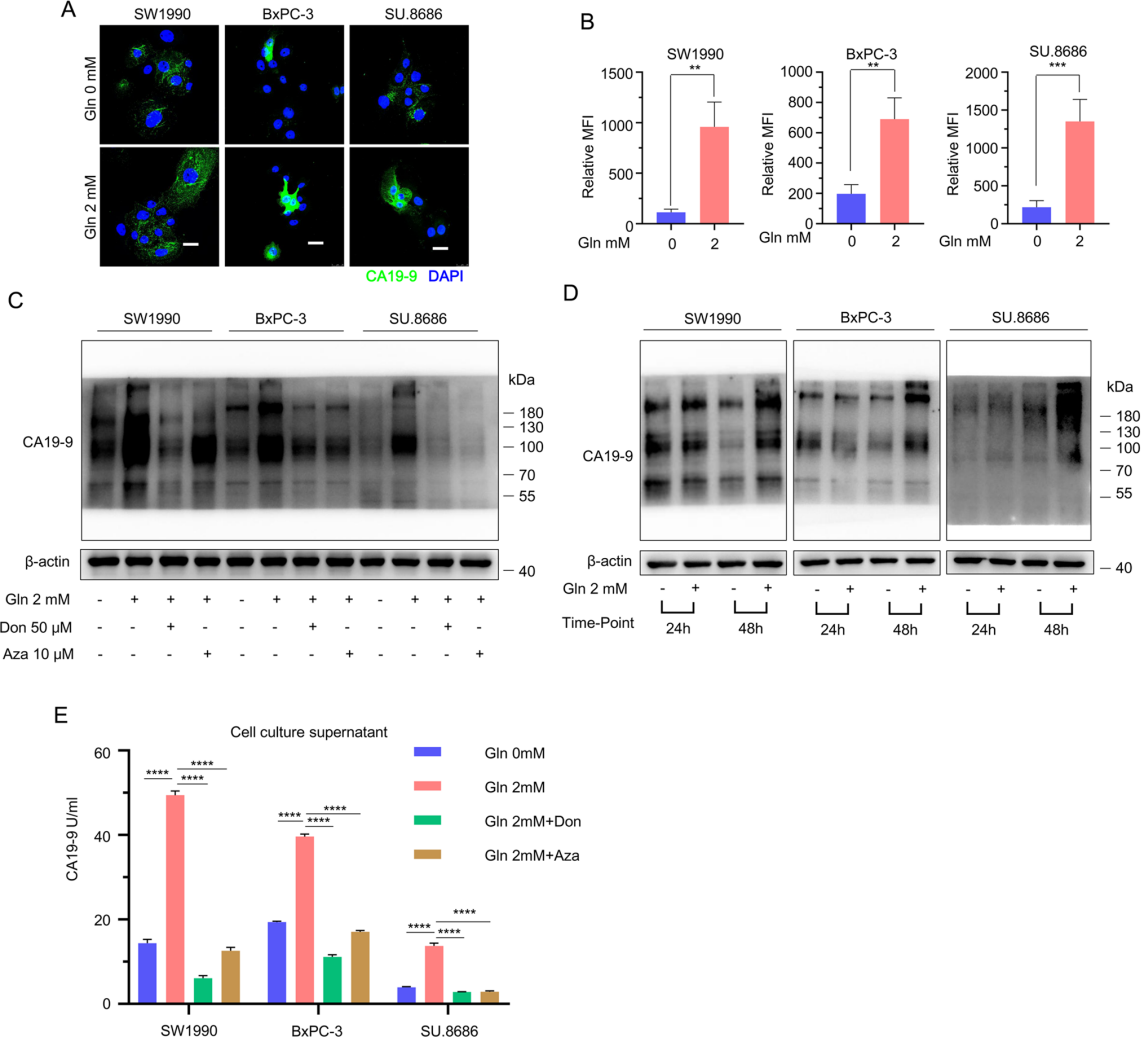
Glutamine promotes CA19-9 biosynthesis in vitro. **A**, **B** The addition of 2 mM glutamine to glutamine-free medium increased the expression of CA19-9 in SW1990, BxPC-3, and SU.8686 cells, as observed by immunofluorescence confocal microscopy. Cells were cultured in different media for 72 h. Quantification is shown to the right. Data were randomly chosen in seven different fields. Scale bars, 25 μm. **C** The expression of CA19-9 in cells cultured for 72 h in glutamine-free medium, 2 mM glutamine medium, and 2 mM glutamine medium treated with 50 µM Don or 10 µM Aza was examined by Western blot. **D** The expression of CA19-9 in pancreatic cancer cells cultured in glutamine-free medium or 2 mM glutamine medium for 24 or 48 h was examined by western blot. **E** The concentration of CA19-9 in the supernatants of cells cultured for 72 h in glutamine-free medium, 2 mM glutamine medium, and 2 mM glutamine medium treated with 50 µM Don or 10 µM Aza was examined by electrochemiluminescence immunoassay. Three replicates per condition. All data are presented as the mean ± s.e.m. Three replicates per condition. *Don* diazooxonorleucine, *Aza* azaserine, *UHPLC-MS* ultrahigh performance liquid chromatography coupled with tandem Q-Exactive Orbitrap mass spectrometer. Data represent the mean ± s.e.m. *p < 0.05; **p < 0.01; ***p < 0.001; ****p < 0.0001. *ns* not significant

CA19-9 is the most widely used biomarker for pancreatic cancer [5, 20]. Accordingly, understanding the biosynthesis of CA19-9 may provide a potential solution for the treatment of pancreatic cancer. To confirm the connection between glutamine and CA19-9 biosynthesis, the impact of glutamine on CA19-9 biosynthesis was evaluated in vitro and in clinical samples. Glutamine supplementation increased the levels of CA19-9 in SW1990, BxPC-3, and SU.8686 cells (Fig. 6A, C) and their supernatant (Fig. 6E) cultured for 72 h. The effect of glutamine promoting CA19-9 biosynthesis was also present when pancreatic cancer cells were cultured for 48 h (Fig. 6D). Don and azaserine, two glutaminase inhibitors, decreased the level of CA19-9 in pancreatic cancer cells (Fig. 6C, E).

**Fig. 7:**
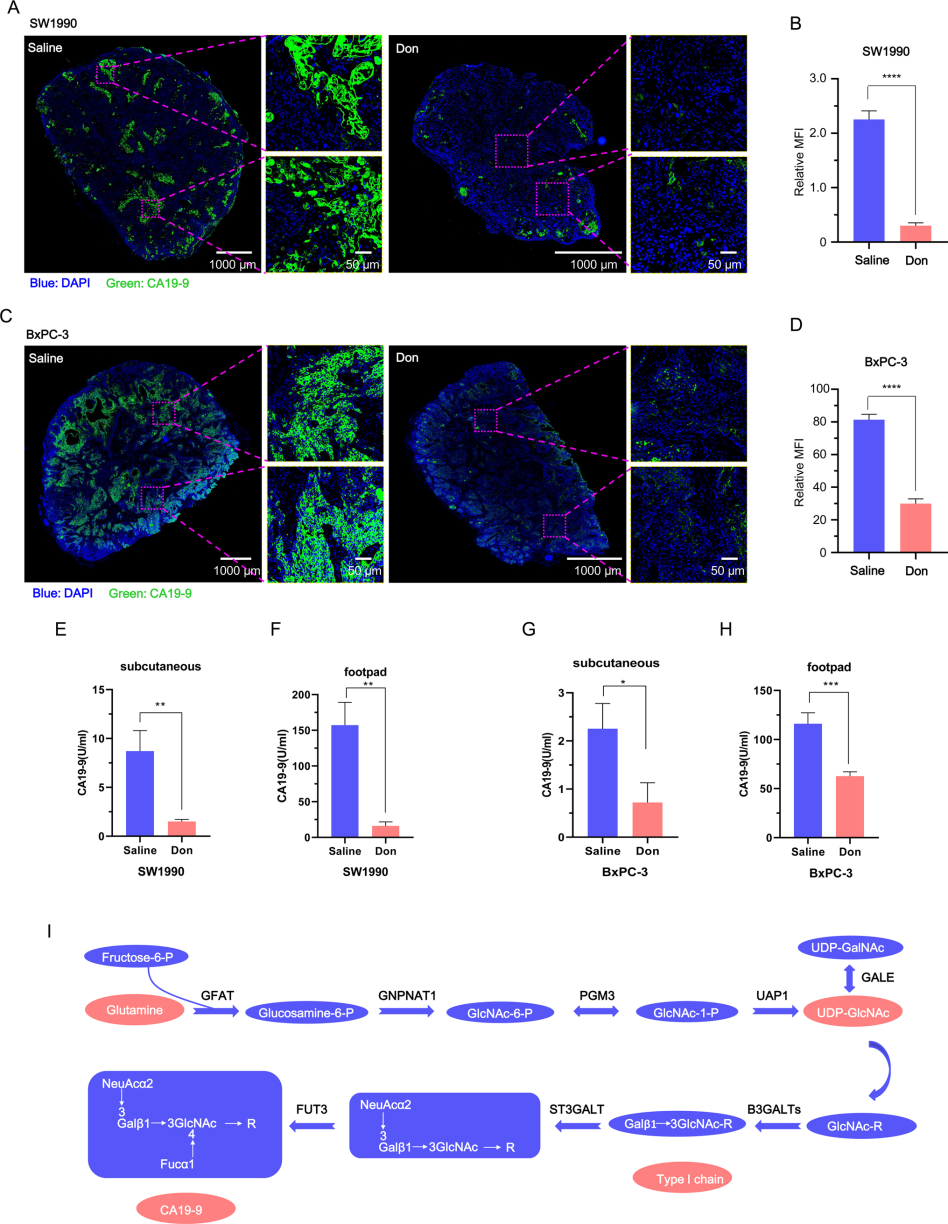
Glutamine promotes CA19-9 biosynthesis in vivo. **A-D** The expression of CA19-9 in SW1990 (**A**) and BxPC-3 (**C**) subcutaneous tumor models was examined by immunofluorescence confocal microscopy. Mice were treated with or without Don. Quantification is shown to the right (**B**, **D**). Data are randomly chosen in seven different fields. **E**, **F** The serum concentration of CA19-9 for SW1990 subcutaneous and footpad animal models by electrochemiluminescence immunoassay. Animals were treated with or without 15 μg/g Don. n = 6 mice per group for subcutaneous models and n = 5 mice per group for footpad models. **G**, **H** The serum concentration of CA19-9 for BxPC-3 subcutaneous and footpad animal models by electrochemiluminescence immunoassay. Animals were treated with or without 15 μg/g Don. n = 8 mice per group. **I** Schematic depicting the synthesis of CA19-9 from glutamine. UDP-GlcNAc is an intermediate product for CA19-9 biosynthesis. The double arrow represents multiple reactions. *UDP-GlcNAc* UDP-N-acetylglucosamine, *Gln* glutamine, *Don* Diazooxonorleucine, *Aza* Azaserine. Data represent mean ± s.e.m. *p < 0.05; **p < 0.01; ***p < 0.001; ****p < 0.0001. *ns* not significant

To examine the impact of glutamine blockade on CA19-9 levels, both subcutaneous and footpad mice models established by SW1990 and BxPC-3 cells were used. Tumor content of CA19-9 was evaluated by immunofluorescence and serum concentration of CA19-9 was examined by electrochemiluminescence immunoassay. Don inhibited tissue (Fig. 7A–D) and serum content (Fig. 7E–H) of CA19-9 in subcutaneous and footpad mice models. Since the type I chain (Gal-GlcNAc) is the backbone structure of CA19-9 [6], we propose that glutamine is a substrate for CA19-9 biosynthesis and UDP-GlcNAc, the end-product of the hexosamine biosynthetic pathway, is an intermediate product between glutamine and CA19-9 (Fig. 7I).

## Discussion

Pancreatic cancer cells have a strong dependence on glutamine for growth and metastasis [11, 21]. In this study, we demonstrated that glutamine was rich in highly aggressive and glycosylated pancreatic cancers. The glutamine concentration in clinical tissues was associated with liver metastasis or lymphovascular invasion. Glutamine promotes O-GlcNAcylation, protein glycosylation, and CA19-9 biosynthesis through the hexosamine biosynthetic pathway. Thus, glutamine promotes pancreatic cancer progression and O-GlcNAcylation (Fig. 7I).

Our study suggests that glutamine promotes tumor progression and CA19-9 biosynthesis in pancreatic cancer. Overall, glutamine promotes pancreatic cancer progression by ATP generation, biomass production, and signal transduction [10]. It is a major source of energy and nitrogen for biosynthesis and a carbon substrate for anabolic processes in cancer cells. However, the role of glutamine in signal transduction has largely been neglected. In this study, we found that glutamine can enhance CA19-9 production and glycosylation. Taken together, glutamine plays a comprehensive role in pancreatic cancer progression.

The concept of glutamine restriction for the treatment of cancers has existed for a long time [9, 11]. The translational value for pancreatic cancer is strengthened by our study, which connects glutamine with CA19-9 biosynthesis. Dietary modification of the host has a major impact on the availability of glutamine in the tumor microenvironment and thereby the progression of pancreatic cancer [22]. Importantly, glutamine limitation may have a synergistic effect on other therapeutic methods. For example, a glutamine restriction diet has been reported to improve the survival of medulloblastoma animal models, and this effect could be strengthened when combined with cisplatin chemotherapy [23].

Although our report suggests that glutamine promotes pancreatic cancer progression and CA19-9 biosynthesis, a therapeutic strategy involving glutamine restriction should be administered with caution. First, glutamine is an important nutrient for the human body, which limits the degree of glutamine reduction. Clinical trials utilizing Don have been abandoned for its serious side effects, especially unacceptable gastrointestinal toxicities [24]. In addition, perturbation of glutamine metabolism may provoke systemic metabolic compensation, which could have a complex effect on cancer progression [25]. For example, pancreatic cancer cells rescue the glutamine deprivation crisis by de novo biosynthesis using α-ketoglutarate (α-KG) from the TCA cycle, which is mediated by glutamate-ammonia ligase (*GLUL*) [14]. Therefore, balancing treatments and side effects and controlling intrinsic glutamine biosynthesis are the major tasks for the development of glutamine inhibitors in cancer management.

Pancreatic cancer exhibits extremely abundant desmoplasia, which is mainly derived from pancreatic stellate cells [3]. Pancreatic cancer cells usually reside in a barren microenvironment where nutrients and oxygen are scarce. One previous study showed that stroma served as a proline reservoir for glutamine and other nutrient production under nutrient deprivation conditions [26]. The interconversion among glutamine, proline, and collagen is mainly mediated by proline dehydrogenase 1 (PRODH1), which is necessary for pancreatic cancer cell growth. Our study indicates that a potential interconversion exists between glutamine and proline/stroma in pancreatic cancer. However, whether the abundant stroma promotes pancreatic cancer progression by supplying glutamine for tumor growth and CA19-9 production needs further study.

In conclusion, the above findings suggest that pancreatic cancer cells, which are different from their nontransformed counterparts, have a strong metabolic dependency on glutamine. Thus, glutamine addiction may serve as an appealing vulnerability for the treatment of pancreatic cancer.

## Materials and methods

### Patient data collection and ethics statement

Medical records were retrieved from a database constructed by the Fudan University Shanghai Cancer Center. Variables including age, sex, tumor location, metastatic status, size, CA19-9, lymphatic metastasis, and overall survival were retrieved. Pancreatic cancers were classified according to the 8^th^ American Joint Committee on Cancer (AJCC) staging system. All pancreatic cancer tissue specimens were obtained from the Fudan University Shanghai Cancer Center, Shanghai, China. All patients were given informed consent, which was consented by the Institutional Review Board of the Fudan University Shanghai Cancer Center.

### Animal protocols, diets, and treatment

Six-week-old athymic BALB/c nude mice (female) were obtained from Shanghai Jihui Laboratory Animal Co., Ltd. (Shanghai, China) and maintained under special pathogen-free (SPF) facilities with approved experimental protocols. None of the experiments exceeded the limits (2000 mm^3^) permitted by the IACUC of Fudan University. Experiments were performed in accordance with the institutional ethical guidelines and were approved by the institutional review board of Fudan University.

### Cell lines and cell culture

BxPC-3, SU.8686, and SW1990 cells were purchased from American Type Culture Collection (ATCC). All the cells were kept in DMEM with 4.5 g/liter glucose supplemented with 10% FBS and 1% penicillin/streptomycin (Gibco; Thermo Fisher Scientific, Inc.). Cell lines were maintained in a 5% CO_2_ incubator at 37 °C. Cells used for the experimental study were passaged within 10 to 15 passages after reviving from the frozen vials. Cell lines were routinely tested to exclude Mycoplasma contamination. Cell proliferation was evaluated every 24 h using a Cell Counting Kit-8 (DOJINDO, Japan), according to the manufacturer’s instructions.

### Immunofluorescence staining

For cell staining, cells grown on coverslips were fixed with 4% paraformaldehyde and permeabilized with 0.1% Triton X-100 in TBS (50 mM Tris–HCl, pH 7.4, 150 mM NaCl) buffer. For specimen staining, formalin-fixed, paraffin-embedded sections of pancreatic cancer tissues were obtained. After blocking with 5% BSA (Yeasen, Shanghai) in PBS, antibodies against O-GlcNAc (Abcam, Inc., ab2739) or CA19-9 (Abcam, Inc., ab3982) at a dilution of 1/200 in 5% BSA were used to detect their expression at 4 °C overnight. After washing, the samples were stained with AlexaFluor^®^ 488 goat anti-rabbit secondary antibody (Jackson ImmunoResearch, Inc.) or AlexaFluor^®^ 594 goat anti-mouse secondary antibody (Jackson ImmunoResearch, Inc.) at a 1/200 dilution for 1 h at room temperature. DAPI (Southern Biotech, Inc.) was used as the nuclear counterstain. Fluorescent images were acquired using a Leica confocal laser scanning microscope (Leica, Inc.). Confocal imaging was performed in seven random fields. Mean fluorescence intensity (MFI) was calculated using Image-Pro plus software (version 6.0, Media Cybernetics, Inc.).

### Western blot and lectin blot analyses

Collected cells were incubated in RIPA buffer, 1 mM PMSF, and 1× Proteinase Inhibitor Cocktail (Beyotime, China) for 30 min. Diluted protein samples were added to 5 × SDS‒PAGE loading buffer and then heated at 100 °C for 10 min to denature proteins. Protein samples (10 μg) were loaded on a 10% SDS‒PAGE gel and run at 100 V for 80 min in SDS‒PAGE running buffer. After blocking with 5% skimmed milk in TBST, the blots were incubated with anti-CA19-9 antibody (Abcam, USA) in blocking solution on a shaker at 4 °C overnight. Blots were incubated with goat anti-mouse IgG (H + L)-HRP or goat anti-rabbit IgG (H + L)-HRP (Proteintech Group, USA) at 1:5,000 in TBST on a shaker at room temperature for 1 h.

For lectin blot analysis, biotinylated *Aleuria aurantia lentin* (AAL, 3 µg/ml), biotinylated *Datura sodium lectin* (DSL, 3 µg/ml), biotinylated *Galanthus nivalis lectin* (GNL, 3 µg/ml), biotinylated *Phaseolus vulgaris leucoagglutinin* (PHA-L, 3 µg/ml), biotinylated *Phaseolus vulgaris erythroagglutinin* (PHA-E, 3 µg/ml), and biotinylated *wheat germ agglutinin* (WGA, 5 µg/ml; Vector Laboratories, Inc.) were incubated with 3% BSA on a shaker for 30 min and then incubated with 0.1 µg/ml streptavidin-HRP conjugate (Vector Laboratories, Inc.) in blocking buffer for 20 min at room temperature [27]. Images were taken using a ChemiScope 3000 mini (CLiNX, Shanghai, China) after the Chemi Signal ECL Plus (CLiNX, China) reaction. The intensity of the blotting signals was measured by densitometric analysis using ImageJ.

### Measurement of CA19-9 in fluids

For patients with pancreatic cancer, serum was collected at the time of diagnosis without major treatments. For mouse models, serum was collected at sacrifice. For cultured cells, supernatants were collected at predetermined times of cell culture. The concentration of CA19-9 in fluids was measured using an electrochemiluminescence immunoassay with a CA19-9 assay kit (Roche) on a Roche Cobase 8000 immunoassay analyzer (Roche Diagnostics, Mannheim, Germany) [28].

### Subcutaneous animal models

The right flanks of mice were injected subcutaneously with 1 × 10^6^ pancreatic cancer cells. Mice were randomly assigned to the Don group (750 mg/kg) or the control group (the same volume of saline). Drugs were administered intraperitoneally twice weekly after inoculation. Mice were observed twice weekly after inoculation. The size of xenografts was measured twice a week by measuring tumors with calipers. Tumor volumes were determined using the formula ${\text{volume }} = \, \left( {\text{long axis}} \right) \, \times \, \left( {\text{short axis}} \right)^{{2}} \times \, \pi /{6}$. At the indicated times, the tumors were surgically dissected and weighed. Samples were then processed for histopathologic examination.

### Lymphatic metastasis models

The right nail pad of mice was injected subcutaneously with 1×10^6^ pancreatic cancer cells [29]. Mice were randomly assigned to the Don group (750 mg/kg) or the control group (the same volume of saline). Drugs were administered intraperitoneally twice weekly after inoculation. Mice were observed twice weekly after inoculation. At the indicated times, the animals were euthanized, and the popliteal, inguinal, iliac and renal hilus lymph nodes were harvested to examine lymphatic metastasis. Metastatic lymph nodes were confirmed by H&E staining and pathological examination.

### Intraperitoneal mouse models

Mice were injected intraperitoneally with 1×10^6^ pancreatic cancer cells. Mice were randomly assigned to the Don group (750 mg/kg) or the control group (the same volume of saline). Drugs were administered intraperitoneally twice weekly after inoculation. Mice were observed twice weekly after inoculation. At the indicated times, the animals were euthanized, and the intraperitoneal metastases were harvested. Intraperitoneal implantation was confirmed by H&E staining and pathological examination.

### UHPLC‒MS-based quantitative measurement of CA19-9

SU. 8686 and SW1990 pancreatic cancer cells were cultured for 72 h in L-glutamine-^15^N_2_ (Sigma‒Aldrich, 490032). Cells cultured in unlabeled glutamine (Sigma‒Aldrich, Cat.# G3126) were used as controls. A total of 1 × 10^7^ cells were used for analysis. Before examination, a standard curve was built using 3'-Sialyl Lewis A (Carbosynth, Cat.# OS00745) diluted in methanol/water (1:1) at different concentrations (1, 5, 10, 50, 100, 500, 1000, 5000, 10000 nM). A total of 160 μL of methanol (precooled at − 20 °C) was added to each sample for metabolite extraction. Then, the samples were centrifuged at 18000 g and 4 °C for 20 min, and the clear supernatant was transferred to an autosampler vial for UHPLC‒MS/MS analysis. Ultra-performance liquid chromatography coupled to a tandem mass spectrometry system (ACQUITY UPLC I class-Xevo TQ-S system, Waters Corp., Milford, MA, USA) equipped with a Waters ACQUITY UPLC BEH Amide column (100 × 2.1 mm, 1.7 μm) was used to measure the metabolites. Mobile phase A was 10 mM ammonium acetate in water, and mobile phase B was acetonitrile. The elution gradient was set as follows: 0–1.0 min, 1% A; 1.0–3.0 min, 1–40% A; 3.0–5.0 min, 40% A; 5.0–5.1 min, 40–1% A; 5.1–6 min, 1% A. The injected volume was 5 μL. Data acquisition and metabolite quantification were performed using MassLynx 4.1 software (Waters Corp., Milford, MA, USA).

### Label-free quantitative N-glycosylation proteomics

Proteins were extracted using SDT lysis buffer (4% SDS, 100 mM DTT, 100 mM Tris–HCl pH 8.0). Protein digestion was carried out using the FASP method described by Wisniewski et al. [30]. Four hundred micrograms of tryptic peptides was transferred to YM-30 filtration units and incubated for 1 h with a lectin mixture containing 90 µg ConA, 90 µg WGA and 90 µg RCA120 in 36 µl 2X binding buffer [31, 32]. LC‒MS/MS was performed on a Q Exactive Plus mass spectrometer coupled with Easy 1200 nLC (Thermo Fisher Scientific). The MS data were analyzed using MaxQuant software version 1.6.1.0. MS data were searched against the SwissProt human database (20431 total entries, downloaded 10/15/2019). Construction of protein–protein interaction (PPI) networks was also performed using the STRING database with Cytoscape software [33]. The Motif-X algorithm in the MEME Suite (MEME version 5.1.0) was used for N-linked glycosylation motif analysis [34].

### Ultrahigh-performance liquid chromatography (UHPLC)-MS/MS

For metabolite extraction, 1000 μL of extract solvent (precooled at − 20 °C, acetonitrile-methanol–water, 2:2:1) was added to each sample, and the samples were vortexed for 30 s, homogenized at 45 Hz for 4 min, and sonicated for 5 min in an ice-water bath. UHPLC separation was performed using an Agilent 1290 Infinity II series UHPLC System (Agilent Technologies, Inc., Santa Clara, CA, USA) equipped with a Waters ACQUITY UPLC BEH Amide column (100 × 2.1 mm, 1.7 μm). An Agilent 6460 triple quadrupole mass spectrometer (Agilent Technologies, Inc., Santa Clara, CA, USA) equipped with an AJS electrospray ionization (AJS-ESI) interface was used for assay development. Agilent MassHunter Work Station Software (B.08.00, Agilent Technologies) was applied for MRM data acquisition and processing.

### Isotope tracing untargeted metabolomics

SU. 8686 pancreatic cancer cells were cultured for 12 h or 24 h in L-glutamine-^13^C_5_ (2 mM, Cat. #605166, Sigma‒Aldrich) or L-glutamine-^15^N_2_ (Sigma‒Aldrich, 490032). Cells cultured in unlabeled glutamine (Sigma‒Aldrich, Cat.# G3126) were used as controls. A total of 1 × 10^7^ cells were sent for analysis. Samples were prepared as previously described [35]. LC‒MS/MS analyses were performed using a UHPLC system (1290, Agilent Technologies) with a UPLC HSS T3 column (2.1 mm × 100 mm, 1.8 μm) coupled to a Q Exactive mass spectrometer (Thermo). Mobile phase A was 0.1% formic acid in water for positive mode and 5 mmol/L ammonium acetate in water for negative mode, and mobile phase B was acetonitrile. The elution gradient was set as follows: 0–1.0 min, 1% B; 1.0–8.0 min, 1–99% B; 8.0–10.0 min, 99% B; 10.0–10.1 min, 99–1% B; 10.1–12 min, 1% B. The flow rate was 0.5 mL/min. The raw data were converted to the mzXML format using ProteoWizard and processed with an in-house program, which was developed using R and based on XCMS, for peak detection, extraction, alignment, and integration [36]. Then, an in-house MS2 database (BiotreeDB) was applied for metabolite annotation. The cutoff for annotation was set at 0.3.

### Statistical analysis

The statistical analyses were performed using SPSS 19.0 software (IBM Corp.) and Prism statistical software (version 8, GraphPad Software, Inc.). Paired 2-tailed Student’s t tests were used to examine the difference in glutamine concentration between primary pancreatic cancers and their matched metastases (liver or lymph node). Unpaired 2-tailed Student’s t tests were used to evaluate other data. For multiple comparisons, ANOVA combined with Bonferroni was applied. Data are presented as the mean ± standard error of the mean. Spearman correlation analysis was used to determine the correlation between glutamine concentration and other variables. Survival analysis was calculated by the Kaplan–Meier method, and the survival curves were examined by log-rank tests. Significance was defined as a p value < 0.05, unless otherwise stated.

## Data Availability

All other data needed to evaluate the conclusions in the paper are present in the paper. Data are available on request from the corresponding author.

## Abbreviations

Aza: Azaserine; CA19-9: carbohydrate antigen 19–9
coIP-MS: Co-immunoprecipitation mass spectrometry
Don: Diazooxonorleucine
GLUL: Glutamate-ammonia ligase
HBP: Hexosamine biosynthetic pathway
KEGG: Kyoto encyclopedia of genes and genomes
O-GlcNAc: O-linked n-acetylglucosamine
PDAC: Pancreatic ductal adenocarcinoma
PRODH: Proline dehydrogenase 1
UDP-GalNAc: UDP-n-acetyl-D-galactosamine
UDP-GlcNAc: UDP-n-acetylglucosamine
UHPLC–MS/MS: Ultra-high performance liquid chromatography tandem mass spectrometry
